# Religiosity, Cognition, and Engagement in Advance Care Planning Among Older Adults

**DOI:** 10.1093/geroni/igaa057.1353

**Published:** 2020-12-16

**Authors:** Richard Chunga, Jeffrey Stokes, Beth Dugan

**Affiliations:** University of Massachusetts Boston, Boston, Massachusetts, United States

## Abstract

Advance care planning (ACP) is an important process of discussion and documentation that may help older adults receive the end-of-life care they prefer. Although existing literature predominantly finds greater self-reported religiosity to decrease the rates of ACP, a clear consensus is not yet evident. Data from 3,182 adults aged 55 and older participating in the 2012 wave of the Health and Retirement Study were used to investigate this association and examine the moderating role of cognition. Participants reporting at least one of two ACP behaviors (written instructions and assigning a health care proxy) were categorized as formal planning only, engaging in only informal discussions was categorized as informal planning, and those who completed both or none were categorized accordingly. Cognition was measured using self-reported memory change over the last two years and with objective cognitive testing scores. Using multinomial logistic regression, three forms of ACP behaviors were regressed on a religiosity/spirituality scale, the two cognition measures, and demographic and psychosocial covariates. Greater religiosity was associated with a lower likelihood of engaging in both plans compared to none (OR=0.91, 95%CI=0.84-0.97), however this effect was no longer significant with the inclusion of race. Higher cognitive scores were associated with greater odds of engaging in informal-only (OR=1.07, 95%CI=1.04-1.10) and both plans (OR=1.04, 95%CI=1.01-1.06); subjective memory change was not associated with ACP. Neither cognitive measure significantly moderated the negative association of religiosity on ACP, suggesting that the awareness of worsening memory does not undermine the tendency to avoid planning among the highly religious.

